# Somatostatin Receptor 2 Negative Pheochromocytoma Masked by Normal Adrenal Gland on Gallium-68 DOTATATE

**DOI:** 10.1016/j.aace.2024.12.011

**Published:** 2024-12-28

**Authors:** Sanghwa E. Park, Thanh D. Hoang, Derek J. Stocker, Mohamed K.M. Shakir, Andrew J. Spiro

**Affiliations:** 1 Carl R. Darnall Army Medical Center, Fort Cavazos, Texas; 2 Department of Endocrinology, Walter Reed National Military Medical Center, Bethesda, Maryland; 3 Department of Nuclear Medicine, Walter Reed National Military Medical Center, Bethesda, Maryland

**Keywords:** pheochromocytoma, ^68^Ga-DOTATATE PET/CT (^68^Ga-DOTATATE), somatostatin receptor 2 (SSTR2)

## Abstract

**Background/Objective:**

Gallium-68 DOTATATE (^68^Ga-DOTATATE) positron emission tomography/computed tomography (CT) is a somatostatin receptor (SSTR)-based imaging with high sensitivity that can be used for detection of pheochromocytomas and paragangliomas. We report a pheochromocytoma with negative SSTR2 expression and low uptake on ^68^Ga-DOTATATE, whose detection was masked by the uptake of normal adrenal tissue.

**Case Report:**

A 50-year-old man presented with a right adrenal incidentaloma. He had mildly elevated plasma normetanephrine levels of 194 pg/mL (ref. 0-145 pg/mL). Adrenal CT scan showed a right 1.9-cm lesion with unenhanced attenuation of 38 Hounsfield units. ^68^Ga-DOTATATE showed a 1.9-cm right adrenal lesion and reported diffuse uptake in the adrenal glands, with maximum standardized uptake value (SUVmax) of 17.23 on the right and SUVmax of 22.78 on the left. After a 2-year interval, plasma normetanephrine level increased to 420 pg/mL (ref. 0-136.8 pg/mL). Adrenal CT scan showed the right adrenal lesion increased in size to 2.6 cm. He underwent right adrenalectomy, and pathology reported a 2.3-cm pheochromocytoma. Subsequent review of the initial ^68^Ga-DOTATATE identified the pheochromocytoma as a photopenic area in the right adrenal gland with 7.73 SUVmax. Tissue staining was negative for SSTR2 expression. Genetic testing was negative for pheochromocytoma syndromes.

**Discussion:**

Although ^68^Ga-DOTATATE has strong affinity for SSTR2, some pheochromocytomas have low expression of SSTR2. The negative SSTR2 expression, small lesion size, and background uptake of the adrenal gland can affect the detection of pheochromocytoma.

**Conclusion:**

^68^Ga-DOTATATE may have limitations when evaluating small pheochromocytomas or other neuroendocrine tumors with low SSTR2 expression.

**Highlights**

• Gallium-68 DOTATATE (^68^Ga-DOTATATE) positron emission tomography/computed tomography is somatostatin receptor (SSTR)-based imaging used for the evaluation of neuroendocrine tumors, including pheochromocytomas
• Although ^68^Ga-DOTATATE has strong affinity for SSTR2, some pheochromocytomas have low SSTR2 expression
• Normal adrenal glands demonstrate significant uptake on ^68^Ga-DOTATATE
• Pheochromocytomas with negative SSTR2 expression may have low uptake on ^68^Ga-DOTATATE, and smaller lesions can be masked by normal adrenal glands

**Clinical Relevance**

Gallium-68 DOTATATE (^68^Ga-DOTATATE) is increasingly used for the evaluation of neuroendocrine tumors, including pheochromocytomas. It is important for providers to understand the potential limitations of the imaging technique. We report a pheochromocytoma with negative somatostatin receptor 2 expression and low uptake on ^68^Ga-DOTATATE, whose detection was masked by the uptake of normal adrenal tissue.

## Introduction

Pheochromocytomas are rare catecholamine-producing neuroendocrine tumors (NETs) that form from the chromaffin cells of the adrenal medulla. They can produce excess catecholamines, which can lead to symptoms like palpitations, headache, diaphoresis, and increased blood pressure.^1^ The majority of these tumors are found incidentally on abdominal imaging. Imaging characteristics including attenuation on unenhanced computed tomography (CT), lesion size, laterality, rate of growth, shape, heterogeneity, necrosis, and calcifications help differentiate benign lesions from pheochromocytoma or malignancy.^2^^,^^3^ Unenhanced adrenal CT is considered as first-line imaging for evaluating adrenal masses, with an attenuation value of <10 Hounsfield units (HUs) consistent with lipid-rich, benign lesions. The same can be shown on magnetic resonance imaging (MRI) with chemical-shift analysis. Most pheochromocytomas show an unenhanced attenuation of >20 HU on a CT scan. Fluorodeoxyglucose positron emission tomography (PET)/CT is generally not indicated unless evaluating for metastatic or multifocal disease, and can result in false-positive results with functional adenomas or false-negative results with smaller lesions.^2^ Octreotide scans have traditionally been used for NET imaging, but more recently, gallium-68 DOTA-peptides including ^68^Ga-DOTATATE have been developed for functional imaging.^4^ ^68^Ga-DOTATATE is a somatostatin receptor (SSTR)-based imaging that has high sensitivity for the detection of pheochromocytomas and paragangliomas.^5^^,^^6^ We report the case of a pheochromocytoma with negative SSTR2 expression and resultant low uptake on ^68^Ga-DOTATATE, whose detection was masked by the greater uptake of the normal adrenal tissue surrounding the lesion.

## Case Report

A 50-year-old man was evaluated for a right adrenal nodule found incidentally on a spinal MRI, ordered due to chronic back pain. He did not have hypertension or other chronic medical issues and was not taking any medication. He had no family history of adrenal tumors or hormone disorders. He denied headaches, palpitations, or diaphoresis. His physical examination was unremarkable with normal blood pressure. A low-dose dexamethasone suppression test was normal with cortisol level of 0.6 μg/dL (ref. <1.8 μg/dL). A plasma aldosterone level of 11.6 ng/dL (ref. 0-30 ng/dL) and a renin activity of 0.604 ng/mL/h (ref. 0.167-5.380 ng/mL/h) were in the normal range, with aldosterone-to-renin activity ratio of <20. Plasma metanephrine level was normal at 48 pg/mL (ref. 0-62 pg/mL), with mildly elevated plasma normetanephrine level of 194 pg/mL (ref. 0-145 pg/mL) (Table).^7^ Urine metanephrine level of 171 μg/24 h (ref. 45-290 μg/24 h) and urine normetanephrine level of 421 μg/24 h (ref. 82-500 μg/24 h) were normal. Adrenal CT scan showed a 1.9 × 1.4 × 1.9 cm right adrenal lesion with unenhanced attenuation of 38 HU, and absolute washout of −95.2% at 15 minutes. Adrenal MRI showed a 2.3 × 1.1 × 1.5 cm lesion, with hyperintense T2 signal, hypointense T1 signal, and no signal dropout on out-of-phase imaging. ^68^Ga-DOTATATE showed a 1.9-cm right adrenal lesion and reported diffuse uptake in the adrenal glands, with 17.23 standardized uptake value (SUVmax) on the right and 22.78 SUVmax on the left. The formal read noted “nonspecific PET/CT findings.” A joint decision was made for surveillance of the adrenal lesion.

**Table:** Patient Laboratory and Imaging Results During Initial Presentation and 2-Year Follow-Up

Tests	Initial	2-year follow-up
Plasma metanephrine	48 (0-62 pg/mL)	80.0 (0-88 pg/mL)
Plasma normetanephrine	194 (0-145 pg/mL)	420 (0-136.8 pg/mL)
Urine metanephrine	171 (45-290 μg/24 h)	233 (58-276 μg/24 h)
Urine normetanephrine	421 (82-500 μg/24 h)	822 (156-729 μg/24 h)
Adrenal CT	1.9 × 1.4 × 1.9 cm Unenhanced attenuation: 38 HU Absolute washout: −95.2%	2.0 × 1.9 × 2.6 cm Unenhanced attenuation: 23 HU Absolute washout: −95.8%
Clonidine suppression test: ->Plasma normetanephrine: at baseline—312 pg/mL (ref. 0-136.8 pg/mL) ->Plasma normetanephrine: 3 h after clonidine—319 pg/mL (ref. 0-136.8 pg/mL)		
Clonidine suppression test protocol^7^: Patient given 0.3 mg clonidine (recommended for patients 60-80 kg body weight) at baseline. Plasma normetanephrine levels are measured before clonidine and 3 h after dose. If the plasma normetanephrine suppresses <40% from baseline and is above the normal reference range, results are consistent with a pheochromocytoma.		

Abbreviations: CT = computed tomography; HU = Hounsfield unit.

Normal reference ranges noted in parenthesis.

After a 2-year interval, the patient continued to deny headaches, palpitations, or diaphoresis and remained normotensive. Plasma metanephrine level was normal at 80.8 pg/mL (ref. 0-88.0 pg/mL) and plasma normetanephrine level had increased to 420 pg/mL (ref. 0-136.8 pg/mL). Urine metanephrine level was normal at 233 μg/24 h (ref. 58-276 μg/24 h) and urine normetanephrine level was mildly elevated at 822 μg/24 h (ref. 156-729 μg/24 h). Adrenal CT scan showed the right adrenal lesion had increased in size to 2.0 × 1.9 × 2.6 cm, with unenhanced attenuation of 23 HU, and absolute washout of (−95.8%) at 15 minutes. During a clonidine suppression test, the plasma normetanephrine levels did not suppress, consistent with a pheochromocytoma (Table). The patient was referred for surgery and underwent an uncomplicated laparoscopic right adrenalectomy. Low-dose phenoxybenzamine was started 2 weeks before surgery and not increased because the patient was normotensive. He did not require beta-blockade and was admitted the night before surgery for intravenous hydration. Pathology reported a 2.3 × 1.7 × 1.6 cm pheochromocytoma, limited to the adrenal gland, with absent lymphovascular invasion, and a low mitotic rate (<1/10 high-power field).

After surgical diagnosis of the pheochromocytoma, a review of the initial ^68^Ga-DOTATATE found the pheochromocytoma did have uptake; however, it represented a relatively photopenic area within the right adrenal gland. The pheochromocytoma showed 7.73 SUVmax, less than the 17.23 SUVmax of the adjacent right adrenal tissue (Fig. *A*-*F*). Tissue staining showed that the tumor was negative for expression of SSTR2. The patient underwent genetic analysis for pheochromocytoma syndromes and was negative for Von Hippel Lindau, multiple endocrine neoplasm 1, neurofibromatosis 1, rearranged during transfection sequence variants, succinate dehydrogenase (SDH) genes (SDHA, SDHAF2, SDHB, SDHC, and SDHD), familial pheochromocytoma, and transmembrane protein 127.

**Fig:**
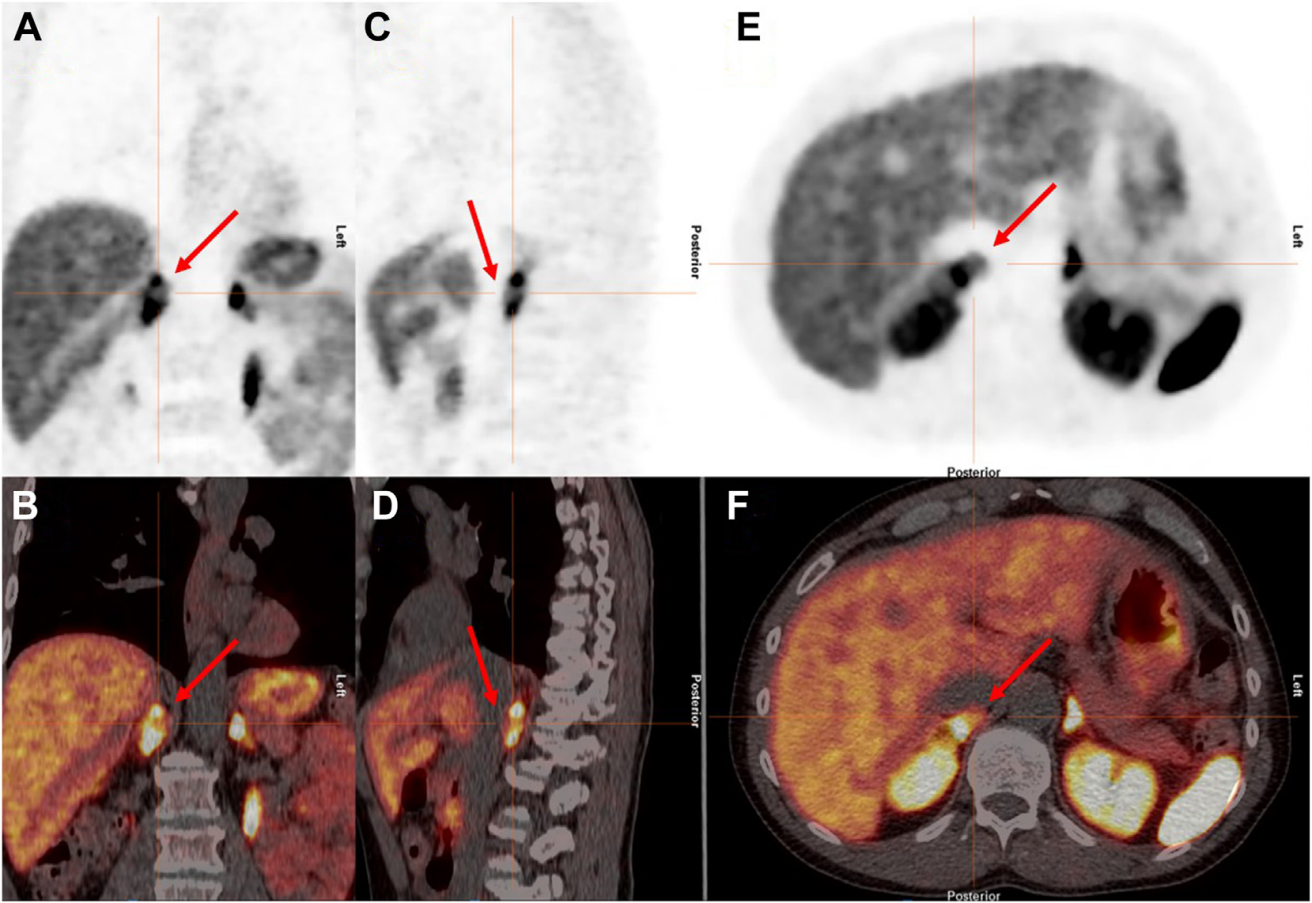
^68^Ga-DOTATATE PET/CT images. *A* and *B,* Coronal view PET. *C* and *D,* Sagittal view PET. *E* and *F*, Transverse view PET. The pheochromocytoma showed maximum standardized uptake value (SUVmax) of 7.73, which was lower than the surrounding normal adrenal tissue SUVmax of 17.23. There is a photopenic area (red arrow) within the right adrenal gland that reflects the location of the pheochromocytoma. *CT* = computed tomography; ^*68*^*Ga-DOTATATE* = Gallium-68 DOTATATE; *PET* = positron emission tomography.

## Discussion

^68^Ga-DOTATATE has been reported to have high sensitivity for the detection of pheochromocytomas; however, in our patient, the findings were nonspecific because the SUVmax of the pheochromocytoma was significantly less than the SUVmax of the normal adrenal tissue. When the ^68^Ga-DOTATATE was initially done, the case was reviewed at an interdisciplinary tumor board. Since the right adrenal gland had less uptake than the left adrenal gland, the findings were considered reassuring that the right adrenal lesion was unlikely to be a pheochromocytoma. During follow-up, the increasing normetanephrine levels and size of the lesion led to renewed suspicion for a pheochromocytoma. There was still doubt though, due to the results of the ^68^Ga-DOTATATE. Hence, the clonidine suppression test was carried out to gain greater confidence that the lesion was a pheochromocytoma, prior to referral to surgery.

Factors that affect how well gallium-based DOTA peptide imaging agents detect lesions include SSTR expression, differentiation, and tumor size.^4^^,^^8^ Our patient’s pheochromocytoma was negative for SSTR2 on tissue staining, which likely contributed to low uptake on ^68^Ga-DOTATATE. SSTRs are G-protein-coupled membrane receptors, and 5 subtypes (SSTR 1-5) are expressed in human tumors.^9^ The majority of NETs express SSTR2 and SSTR5.^10^ ^68^Ga-DOTATATE has strong affinity for SSTR2 and therefore has utility for the characterization of NETs.^8^^,^^11^ Variable rates of SSTR2 expression have been reported in pheochromocytomas.^12^ In one series of 52 pheochromocytomas, 90% expressed SSTR3, whereas only 25% expressed SSTR2. All other SSTR subtypes were expressed at lower rates and about half of the pheochromocytomas expressed more than 1 SSTR.^13^ In another series of 127 pheochromocytomas, 27.6% were SSTR2 negative, whereas 18.1% had intermediate expression and 54.3% had strong expression.^9^ Uptake can also depend on the tumor grade, as low-grade, well-differentiated tumors are better detected by ^68^Ga-DOTATATE, whereas higher grade, dedifferentiated tumors are better detected by 18F-fluorodeoxyglucose.^8^

Another factor that contributed to the pheochromocytoma being missed on ^68^Ga-DOTATATE was the relatively high SUVmax of the adjacent adrenal tissue. One study evaluating the utility of ^68^Ga-DOTATATE compared the SUVmax between pheochromocytomas (n = 24), normal adrenal glands (n = 28), and adrenal adenomas (n = 10). The mean uptake was not significantly different between the pheochromocytomas (30.3 SUVmax) and the normal adrenal glands (18.4 SUVmax). The pheochromocytomas showed greater variability though, with some pheochromocytomas having much less uptake than the adrenal glands. It was also found that the mean uptake in the pheochromocytomas and normal adrenal glands was significantly higher than mean uptake in the adrenal adenomas.^14^

The small size of our patient’s pheochromocytoma and location within the adrenal gland also made it more difficult to identify. The size and location, combined with the low uptake, resulted in a central photopenic defect in the adrenal gland. If the pheochromocytoma had been larger and located on the periphery of the gland, it may have been more apparent, even with the low uptake. There are 2 cases reported of smaller pheochromocytomas, located within highly avid adrenal glands, that produced false-negative results on ^68^Ga-DOTATATE. One interesting difference was that both of those patients had SDHx loss-of-function sequence variants, whereas our patient’s genetic testing was negative. Also, neither of those cases report on the SSTR2 expression of their tumors.^12^^,^^15^ Future direction for improved diagnostics may include use of more targeted PET ligands. For example, ^68^Ga-DOTA-1-NaI3-octreotide, which has affinity for SSTR2, SSTR3, and SSTR5, may have more clearly highlighted our patient’s pheochromocytoma.^11^

## Conclusion

^68^Ga-DOTATATE is an increasingly used type of functional imaging, with utility for evaluation of NETs including pheochromocytomas. ^68^Ga-DOTATATE has a strong affinity for SSTR2, which is expressed in many NETs, but some pheochromocytomas do not express SSTR2 and may not demonstrate strong uptake. With functional imaging, strong uptake in background tissue, like adrenal glands, can also make targets more difficult to identify. The combination of low uptake from negative SSTR2 expression and small lesion size allowed the pheochromocytoma to be masked by normal adrenal tissue. Our case highlights some of the potential pitfalls of functional imaging, specifically the use of ^68^Ga-DOTATATE when evaluating small pheochromocytomas or other NETs that may have low SSTR2 expression.

## Disclosure

The authors have no conflicts of interest to disclose.
